# Sick leave and disability pension in a cohort of TMD-patients – The Swedish National Registry Studies for Surgically Treated TMD (SWEREG-TMD)

**DOI:** 10.1186/s12889-022-13329-z

**Published:** 2022-05-09

**Authors:** Adrian Salinas Fredricson, Carina Krüger Weiner, Johanna Adami, Annika Rosén, Bodil Lund, Britt Hedenberg-Magnusson, Lars Fredriksson, Pia Svedberg, Aron Naimi-Akbar

**Affiliations:** 1 Eastmaninstitutet Department of Oral and Maxillofacial Surgery, Public Dental Services, Folktandvården Stockholm, Stockholm, Sweden; 2 Department of Dental Medicine, Division of Oral Diagnostics and Rehabilitation, Karolinska Institutet, Stockholm, Sweden; 3 Sophiahemmet University, Stockholm, Sweden; 4 Department of Clinical Dentistry, Division of Oral and Maxillofacial Surgery, University of Bergen, Bergen, Norway; 5 Department of Oral and Maxillofacial Surgery, Haukeland University Hospital, Bergen, Norway; 6 Medical Unit for Reconstructive Plastic- and Craniofacial Surgery, Karolinska University Hospital, Stockholm, Sweden; 7 Eastmaninstitutet Department of Orofacial Pain and Jaw Function, Public Dental Services, Folktandvården Stockholm, Stockholm, Sweden; 8 Division of Insurance Medicine, Department of Clinical Neuroscience, Karolinska Institutet, Stockholm, Sweden; 9 Health Technology Assessment-Odontology (HTA-O), Malmö University, Malmö, Sweden

**Keywords:** Temporomandibular disorder, Sick leave, Disability pension, Registry-based research, TMJ surgery, Cohort study

## Abstract

**Background:**

Temporomandibular disorders (TMD) are common and affect approximately 10% of the adult population. TMD is usually associated with headache, pain in the masticatory muscles and/or the temporomandibular joint, clicking or crepitations during mandibular movement as well as painful and/or reduced mouth opening. This study aimed to investigate the level TMD-patients use social insurance benefits before and after their first time of diagnosis or first surgical event, compared to the general population. Furthermore, the aim was to investigate the differences in the use of social insurance benefits between surgically and non-surgically treated TMD-patients that were diagnosed in a hospital setting.

**Methods:**

All Swedish citizens aged 23–59 diagnosed with TMD in a hospital setting and/or surgically treated for the condition during 1998–2016 were identified via the Swedish National Board of Health and Welfare. A non-exposed comparison cohort was collected via the Total Population Registry. Outcome and sociodemographic data were collected via Statistics Sweden. Main outcome was annual net days on sick leave and disability pension five years before (-T5) and five years after (T5) diagnosis and/or surgical treatment (T0). Regression analysis was conducted with generalized estimated equations.

**Results:**

The study included 219 255 individuals (73% female) – 19 934 in the exposed cohort and 199 321 in the comparison cohort. The exposed group was classified into three subgroups: non-surgical, surgically treated once, and surgically treated twice or more. The mean annual net days of sick leave and disability pension combined during the ten-year follow-up was 61 days in the non-surgical group, 76 days in the surgically treated once group, and 104 days in the surgically treated twice or more subgroup. The corresponding number for the non-exposed comparison cohort was 32 days.

**Conclusion:**

Patients diagnosed with TMD in a hospital setting are 2–3 times more dependent on the use of social benefits than the general population. The reliance on sick leave and disability pension is seen as early as five years before diagnosis, and the reliance remains after surgical treatment. The reliance is stronger in patients with several surgical interventions. These findings indicate that patients diagnosed with TMD constitute a patient group with a high burden of health issues causing long-term dependence on social security benefits.

## Background

Temporomandibular disorders (TMD) are relatively common, affecting approximately 10% of the adult general population [1]. The disorders are largely heterogeneous, and the symptoms are most often associated with the muscles in the masticatory system and/or dysfunction in the temporomandibular joint (TMJ) [2]. The symptoms include headache, pain in the masticatory muscles and/or the TMJ, clicking or crepitations during mandibular movement as well as painful and/or reduced mouth opening. The symptoms can appear singular but are often displayed in various combinations.

There are several way to categorize TMD but from a surgical perspective the condition can roughly be divided into three main categories [3];

1. Myofascial pain and dysfunction
2. TMJ functional derangement
3. TMJ degenerative/inflammatory joint disease

Myofascial pain and dysfunction is the predominant type of TMD and these patients are mainly treated conservatively with oral splints or occlusal appliances, jaw movement exercises, behavioural therapy and intra-muscular corticosteroid or local anaesthetic injections [4]. These treatments are often performed by specialists in oral pain and physiology, where the use of Diagnostic Criteria for Temporomandibular Disorders (DC/TMD) as a diagnostic tool is widely applied [5]. In some cases, the treatment is performed by the general dentist. In Sweden, conservative treatment of TMD is conducted within the reimbursement system related to dental care. This is associated with higher expenses for the patients than if they receive treatment in the heavily compensated Swedish public health care system.

A small portion of the patients, less than 1%, are not relieved of their ailments through these non-invasive treatments and may therefore be referred for surgical treatment [6] by the general dentist, the orofacial pain and jaw function specialist or a medical physician. It is however not the magnitude of symptoms, or severity of a certain state, that dictates the choice between surgical or non-surgical treatment. It is the diagnose and/or pathophysiology that form bases for the choice of treatment. For a given condition the least invasive treatment likely to give alleviation should always be first choice. In some TMD conditions, such as myofascial pain, surgery has no place at all regardless of severity. In other cases, such as fibrous or bony ankylosis, conservative treatment is never an option [3]. When patients are referred to an oral and maxillofacial surgeon (OMFS) for surgical treatment they will be subjected to a new evaluation, this time with a more orthopaedic approach, for example by using on or more of the published diagnostic tools [7–10]. The surgical treatment of TMD patients is in Sweden conducted within the public health care system and information regarding the treatment is subsequently reported in the National Patient Registry (NPR).

The two main surgical procedures performed in Sweden is arthroscopy and discectomy, and the efficacy of these methods was first reported during the 1990’s [11, 12], which had a large impact on the surgical treatment options of these patients. From approximately 2015 and forward there was a slow shift from open joint surgery towards more arthroscopic procedures. Less frequent surgical procedures have been gap-osteotomy, coronoidectomy, total joint replacement and other types of arthroplastic surgery. These are expected to be rather stable over time but with a slow shift from gap-surgery to autologous or alloplastic replacement.

It is well-known that the conditions within the TMD spectrum affect one’s ability to perform day-to-day activities, negatively impacting one’s overall quality of life [13]. However, it is unknown how TMD affects work capacity and the reliance on social benefits such as sick leave (SL) and disability pension (DP).

Epidemiological registry-based research on patients with TMD are scarce, and there is a clear lack of knowledge regarding SL and DP among TMD patients. This registry-based retrospective cohort study, that is part of the Swedish National Registry Studies for Surgically Treated TMD (SWEREG-TMD), therefore aims to examine the use of SL and DP in TMD-patients diagnosed and/or surgically treated in a hospital setting in Sweden between 1998 and 2016. The study also aims to investigate the reliance both before and after first time of diagnosis and/or surgical treatment to provide more knowledge regarding reliance on social security benefits that to some extent reflects the severity of disease, but more importantly work incapacity. In addition, this study compares the use of SL and/or DP between patients diagnosed with TMD in a hospital setting but not treated surgically, patients surgically treated and the general population.

## Methods

### Study design and registries

This registry-based retrospective cohort study used data gathered from three nationwide Swedish registries:

1. The NPR, introduced in 1964, was used to collect data defining the exposed cohort. The NPR has a positive predictive value between 85 and 95% and includes data on both in- and out-patient care. The NPR is administered by the National Board of Health and Welfare (NBHW) [14].
2. The Total Population Registry (TPR), introduced in 1968, was used to collect the non-exposed matched comparison cohort and has 100% coverage on all births and deaths in Sweden. The TPR is administered by Statistics Sweden [15].
3. The Longitudinal Integrated Database for Health Insurance and Labour Market Studies (LISA) was used to collect the main outcome variables SL and DP which is prospectively/annually registered in the registry. It was also used to collect sociodemographic covariates of interest. LISA is administered by Statistics Sweden (SCB) [16].

Study subjects were collected from the entire Swedish population between 1998 and 2016, and outcome variables were collected between 1994 and 2017.

### Study population and data collection

Two cohorts were followed in this study. The exposed cohort was compiled of TMD-patients registered in the NPR between 1998 and 2016. The study subjects were considered exposed if they had received an ICD-10 diagnosis for temporomandibular joint disorders (K07.6) during the inclusion time, or if they had received a treatment code corresponding to treatments exclusively given to patients with TMD. The following treatment codes were used as proxies to define TMD exposure: TMJ-disc surgical procedure (EGB10); TMJ arthroscopy (EGA00); TMJ condylectomy (EGB00); TMJ prosthesis surgical procedure (EGC30); other surgical reconstruction of TMJ (EGC99); TMJ synovectomy (EGB20); TMJ biopsy (EGA20); injection of diagnostic or therapeutic substance in the TMJ (TEG10); TMJ condylotomy (EDC00); open reposition of TMJ luxation (EGC00); TMJ plastic surgery (EGC10); and TMJ plastic surgery with bone graft or other type of transplant (EGC20). Although injection of diagnostic or therapeutic substance in the TMJ (TEG10) is a minimally invasive treatment, it was nevertheless considered to be an invasive treatment and therefore used as a proxy for TMD surgical treatment.

As the surgical treatment is conducted within the public health care system the treatment codes are automatically registered in the NPR. Patients may also receive a K07.6 diagnosis within the hospital care system, either by a physician or an OMFS, and if there is no subsequent surgical treatment registered it can be assumed that they thereafter receive treatment for their condition outside the health care system. The ICD 10 diagnose code K07.6 is a general denomination general for “Temporomandibular joint disorders” (TMJD), which in a wider view can be considered to also encompass TMD. Recruitment of the exposed cohort was conducted during 1998–2016 using the 10^th^ revision of International Classification of Diseases (ICD-10). Only hospital visits where K07.6 was set as main diagnosis was used. Since 1997 surgical day care procedures are reported to the NBHW and validation studies have reported high sensitivity for surgical procedures in the NPR, by comparing quality registry data to data available in the NPR [14]. There is currently no Swedish quality registry specific for TMJ surgery.

The exposed group was subclassified into three subgroups: non-surgical, surgically treated once, and surgically treated twice or more. Patients with several surgical procedures registered on the same occasion (*n* = 66), such as bilateral procedures on a single occasion, were considered as surgically treated once. No consideration to the timespan between the surgical interventions was taken when subclassifying the exposed cohort.

The exposed cohort was matched 1:10 to non-exposed subjects from the general population to construct the non-exposed comparison cohort. These subjects were collected from the TPR and were matched for age, sex, living area, and living at the time of the inclusion. All individuals younger than 23 and older than 59 at the time of inclusion were excluded to enable all subjects to contribute five years of outcome data both before and after inclusion.

Linkage between the registries was performed using the Personal Identification Number (PIN), a unique number given to all Swedish citizens [17]. A registry inquiry was sent to NBHW to collect the PINs of all Swedish citizens who would meet the criteria for an exposed subject. The PINs were then used to collect the non-exposed comparison cohort from the TPR. The PINs from both cohorts were after linked to the LISA-registry to collect the outcome variables SL and DP, as well as socio-demographic covariates.

### Outcome variables

In their current forms, the outcome variables SL and DP are available in the Statistics Sweden LISA database from 1994. Because the earliest inclusion into the study were patients from 1998, the study subjects introduced during the first year of the inclusion only contributed four years of data before their inclusion. All Swedish citizens from the age of 16 can receive SL benefits, which is provided by the Swedish Social Insurance Agency. To receive SL benefits, one must have an income from work or unemployment benefits and reduced work capacity as a result of a disease or injury. SL is normally paid by the employer during the first 14 days. After the eighth day, an attestation from a physician is required. DP is available to anyone who has a longstanding or lifelong incapacity to work and is aged 19–64. Both SL and DP are provided by percentages (25, 50, 75, and 100%) of an ordinary work week. In this study, data on SL > 14 days were included, and we used net days for both SL and DP. Net days are the complete full annual days, with partially imbursed time on SL or DP recounted into full days. Outcome variables were collected through 2017 (i.e., not after 31 December 2017).

### Covariates

Educational level was divided into three categories based on the length of the education. Primary and lower secondary school was defined as 0–9 years of education, upper secondary school as 9–12 years, and post-secondary school as more than 12 years (e.g., university studies or other higher education). Country of birth was stratified into four categories: Sweden, Other Nordic countries, European countries, and non-European countries. Eurostat’s Degree of Urbanisation (DEGURBA) classification system was used (revised definition, 2016) to classify living area. Sweden’s 290 municipalities were divided into three subgroups according to DEGURBA: cities/densely populated areas; towns or suburbs/intermediate density areas; and rural areas/sparsely populated areas. These subgroups derive from Statistics Sweden’s publication on regional divisions in Sweden on 1 January 2019 [18, 19]. Sex was classified as a dichotomous variable and men were set as the reference category. Age was divided into the following groups: 23–25, 26–30, 31–35, 36–40, 41–45, 46–50, 51–55, and ≥ 56. Calendar year was used as categorical variable and as an adjustment variable as the overall use of SL and DP varies over time. Time was also remodelled to be set at zero at the year of inclusion (T0) for each individual study subject and its matched non-exposed cohort subjects. This made it possible to follow subjects for five years before inclusion (-T5) and up to five years after inclusion (T5).

### Statistical methods

Number of net days on SL and DP were analysed using generalized estimating equations (GEE) [20]. The outcome variables were modelled as annually repeated measurements. Poisson distribution was assumed for both outcome variables. In the model, the cohort was included as non-exposed (not diagnosed with TMD) and exposed (diagnosed with TMD) with three sub-categories of exposure: non-surgical, surgically treated once, and surgically treated twice or more. Adjustments were made for country of birth, sex, educational level, DEGURBA, age, and calendar year. Calendar year was included as an adjustment variable to handle the change of social security policies over time. All adjustment variables were used as dichotomous dummy variables. Age was used as a categorical variable divided into the intervals previously described. Missing values for the covariates education, country of birth, and DEGURBA were assumed to be missing at random, and Multiple Imputation by Chained Equations (MICE) was used to impute missing data (20 imputations) [21]. Multinomial logistic regression was used to impute DEGURBA and country of birth, whereas ordinal logistical regression was used for educational level.

## Results

### Sample characteristics

**Fig. 1:**
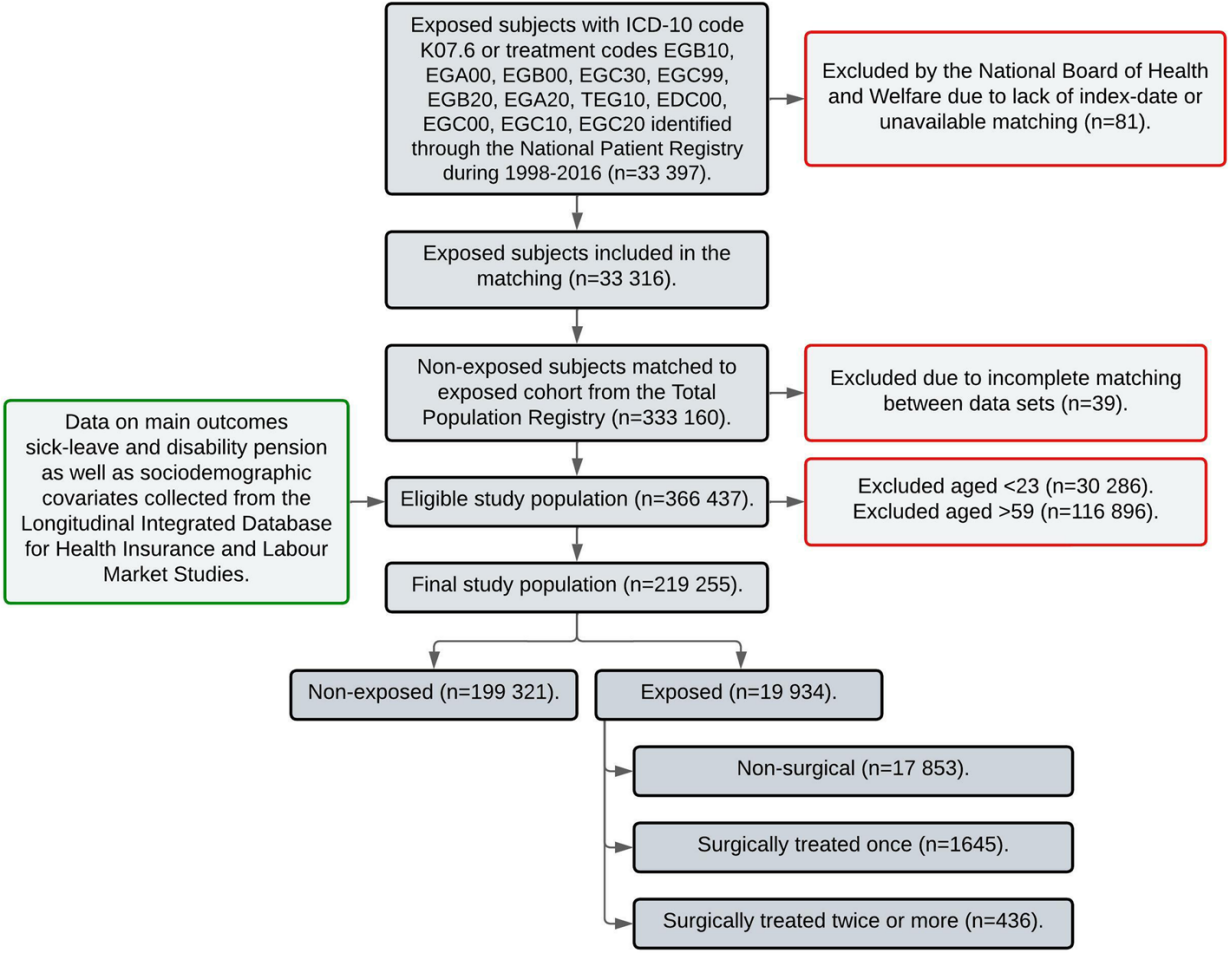
Flow chart describing recruitment of the study subjects and the involved registries. Excluded subjects are shown in boxes highlighted in red

The recruitment and inclusion of the study subjects are displayed in Fig. 1. A total of 33 397 subjects defined as TMD-patients were identified in the NPR [14]. Of these, 81 were excluded by the NBHW as they could not be matched to a non-exposed subject, or a valid index date was missing. Therefore, 33 316 subjects were matched 1:10 to non-exposed individuals through the TPR. During the merging phase, 39 subjects were excluded as we were unable to match them between the datasets. The eligible study population consisted of 366 437 subjects before excluding 147 182 subjects that did not match the age requirements. The final study population consisted of 219 255 individuals, 19 934 exposed and 199 321 non-exposed. The exposed cohort was subdivided into non-surgical (*n* = 17 853), surgically treated once (*n* = 1645) and surgically treated twice or more (*n* = 436).

The attributes and the division of characteristics between the cohorts, age distribution, as well as other baseline covariates are shown in Table 1. The sex division in the entire exposed cohort was 2.6:1. The sex division of the exposed subgroups was greater in the surgically treated groups: the non-surgical subgroup had a female/male ratio of 2.5:1; the surgically treated once subgroup had a ratio of 3.5:1; and the surgically treated twice or more had a ratio of 4.9:1. The mean age was 41.6 years, slightly lower in the surgically treated groups. During the follow-up period, 4291 (2.2%) of the non-exposed study subjects, 385 (2.2%) of the non-surgical subjects, 60 (3.6%) of the surgically treated once subjects, and 18 (4.1%) of the surgically treated twice or more subjects, were deceased.

**Table 1:** Sociodemographic characteristics of the study population

	**General population cohort**	**Exposed cohort**		
		Non-surgical	Surgically treated once	Surgically treated ≥ 2
	*n* = 199 321	*n* = 17 853	*n* = 1645	*n* = 436
**Sex**				
Female	144 542 (73%)	12 817 (72%)	1277 (78%)	362 (83%)
Male	54 779 (27%)	5036 (28%)	368 (22%)	74 (17%)
**Educational level**				
Primary/lower secondary school	23 555 (12%)	2330 (13%)	212 (13%)	61 (14%)
Upper secondary school	84 940 (43%)	8003 (45%)	771 (47%)	215 (49%)
Post-secondary school	88 504 (44%)	7393 (41%)	659 (40%)	159 (37%)
Data unavailable	2322 (1%)	127 (1%)	3 (< 1%)	1 (< 1%)
**Country of birth**				
Sweden	154 467 (78%)	12 845 (72%)	1391 (85%)	383 (88%)
Other Nordic countries	5558 (3%)	465 (3%)	59 (4%)	16 (4%)
Other European countries	16 120 (8%)	1512 (8%)	61 (4%)	14 (3%)
Non-European countries	23 153 (12%)	3030 (17%)	134 (8%)	23 (5%)
Data unavailable	23 (< 1%)	1 (< 1%)	0 (0%)	0 (0%)
**Degree of urbanisation**				
Cities	96 433 (48%)	8319 (47%)	878 (53%)	218 (50%)
Towns and suburbs	51 885 (26%)	5210 (29%)	391 (24%)	120 (28%)
Rural areas	50 044 (25%)	4283 (24%)	372 (23%)	97 (22%)
Data unavailable	959 (< 1%)	41 (< 1%)	4 (< 1%)	1 (< 1%)
**Age**				
Mean	41.63	41.87	39.69	39.36
IQR 25	33	33	30	31
Median	42	42	39	38
IQR 75	51	51	49	48
Range	23–59	23–59	23–59	23–59
**Marital status**				
Married	86 937 (44%)	8128 (46%)	653 (40%)	196 (45%)
Not married	82 317 (41%)	6886 (39%)	746 (45%)	187 (43%)
Divorced	27 038 (14%)	2629 (15%)	228 (14%)	48 (11%)
Widow/widower	1869 (1%)	145 (1%)	11 (1%)	3 (1%)
Other^a^	201 (< 1%)	24 (< 1%)	3 (< 1%)	1 (< 1%)
Data unavailable	959 (< 1%)	41 (< 1%)	4 (< 1%)	1 (< 1%)

Other^a^ = Registered partner, Divorced partner, Surviving partner

The most common surgical treatment was discectomy, followed by arthroscopy. Most patients were only registered as surgically treated on one occasion (*n* = 1645). The second most common number of surgical treatment occasions was two (n = 310). The number of subjects decreased with increasing number of surgeries; during the follow-up, one subject was surgically treated on 12 occasions and received 13 treatment codes, including several arthroscopies, plastic surgeries, and two discectomies.

Follow-up time after inclusion was five years or until the end of 2017. As inclusion was between 1998 and 2016, subjects introduced late in the study were therefore unable to contribute to all five follow-up years. During 2013 and 2016 the number of included subjects was 87 663: 22 139 (10.1%) in 2013; 23 886 (10.9%) in 2014; 21 017 (9.6%) in 2015; and 20 621 (9.4%) in 2016. The number of subjects in each cohort throughout the timespan -T5 to T5 can be found in Table 2. All included subjects contributed with at least one year of follow-up data after inclusion.

**Table 2:** Number of subjects in the cohort throughout the follow-up time. The table also shows crude data on the number of subjects receiving SL or DP of ≥ 90, ≥ 180 and ≥ 270 mean annual days, and mean annual days throughout the complete 10-year follow up time (-T5 to T5) in relation to gender for both SL and DP

	**General population cohort**	**Exposed cohort**		
		Non-surgical	Surgically treated once	Surgically treated ≥ 2
	*n* = 199 321	*n* = 17 853	*n* = 1645	*n* = 436
**Number of subjects in the study population**				
-T5 to T1	199 321	17 853	1645	436
T2	180 575	16 058	1576	425
T3	161 469	14 238	1503	407
T4	139 755	12 203	1393	380
T5	119 629	10 346	1279	338
**Subjects with ≥ 90 mean annual days on SL**				
-T5	6652 (3%)	1044 (6%)	133 (8%)	30 (7%)
-T1	7926 (4%)	1438 (8%)	179 (11%)	67 (15%)
T0	8360 (4%)	1693 (9%)	194 (12%)	66 (15%)
T1	8515 (4%)	1638 (9%)	194 (12%)	81 (19%)
T5	4930 (4%)	743 (7%)	126 (10%)	34 (10%)
**Subjects with ≥ 180 mean annual days on SL**				
-T5	3727 (2%)	590 (3%)	81 (5%)	24 (6%)
-T1	4388 (2%)	877 (5%)	110 (7%)	42 (10%)
T0	4730 (2%)	1081 (6%)	124 (8%)	50 (11%)
T1	4819 (2%)	997 (6%)	122 (7%)	55 (13%)
T5	2732 (2%)	451 (4%)	86 (7%)	15 (4%)
**Subjects with ≥ 270 mean annual days on SL**				
-T5	2064 (1%)	326 (2%)	44 (3%)	9 (2%)
-T1	2450 (1%)	527 (3%)	65 (4%)	26 (6%)
T0	2671 (1%)	633 (4%)	63 (4%)	29 (7%)
T1	2736 (1%)	612 (3%)	75 (5%)	35 (8%)
T5	1532 (1%)	282 (3%)	60 (5%)	9 (3%)
**Mean annual net days SL (-T5 to T5)**				
Men	6.6 days	14.0 days	20.4 days	26.2 days
Women	12.7 days	23.8 days	30.7 days	38.5 days
**Subjects with ≥ 90 mean annual days on DP**				
-T5	10 030 (5%)	1666 (9%)	161 (10%)	69 (17%)
-T1	12 694 (6%)	2161 (12%)	242 (15%)	93 (21%)
T0	13 314 (7%)	2288 (13%)	262 (16%)	99 (23%)
T1	13 890 (7%)	2439 (14%)	287 (17%)	113 (26%)
T5	11 212 (9%)	1993 (19%)	279 (22%)	110 (33%)
**Subjects with ≥ 180 mean annual days on DP**				
-T5	9010 (5%)	1506 (8%)	141 (9%)	60 (15%)
-T1	11 424 (6%)	1951 (11%)	203 (12%)	84 (19%)
T0	11 948 (6%)	2057 (12%)	228 (14%)	89 (20%)
T1	12 485 (6%)	2205 (12%)	252 (15%)	96 (22%)
T5	10 066 (8%)	1802 (17%)	256 (20%)	99 (29%)
**Subjects with ≥ 270 mean annual days on DP**				
-T5	6929 (4%)	1160 (7%)	100 (6%)	42 (10%)
-T1	8939 (4%)	1529 (9%)	158 (10%)	57 (13%)
T0	9400 (5%)	1616 (9%)	169 (10%)	62 (14%)
T1	9855 (5%)	1748 (10%)	193 (12%)	66 (15%)
T5	7813 (7%)	1422 (14%)	198 (15%)	77 (23%)
**Mean annual net days DP (-T5 to T5)**				
Men	15.3 days	27.9 days	39.5 days	53.8 days
Women	22.8 days	44.6 days	49.5 days	70.6 days

### Outcome data

At -T5, the percentage of data missing on SL and DP was 6.23% in the non-exposed group and 4.74% in the exposed cohort. At -T1, the missing data on SL and DP was 0.77% in the non-exposed cohort and 0.40% in the exposed cohort. At T5, missing data on SL and DP was 3.62% in the non-exposed group and 2.37% in the exposed cohort. Crude data on the number of subjects receiving SL or DP of ≥ 90, ≥ 180 and ≥ 270 mean annual days is displayed in Table 2, which also shows the mean annual days throughout the complete 10-year follow up time in relation to gender for both SL and DP.

#### Sick leave

**Fig. 2:**
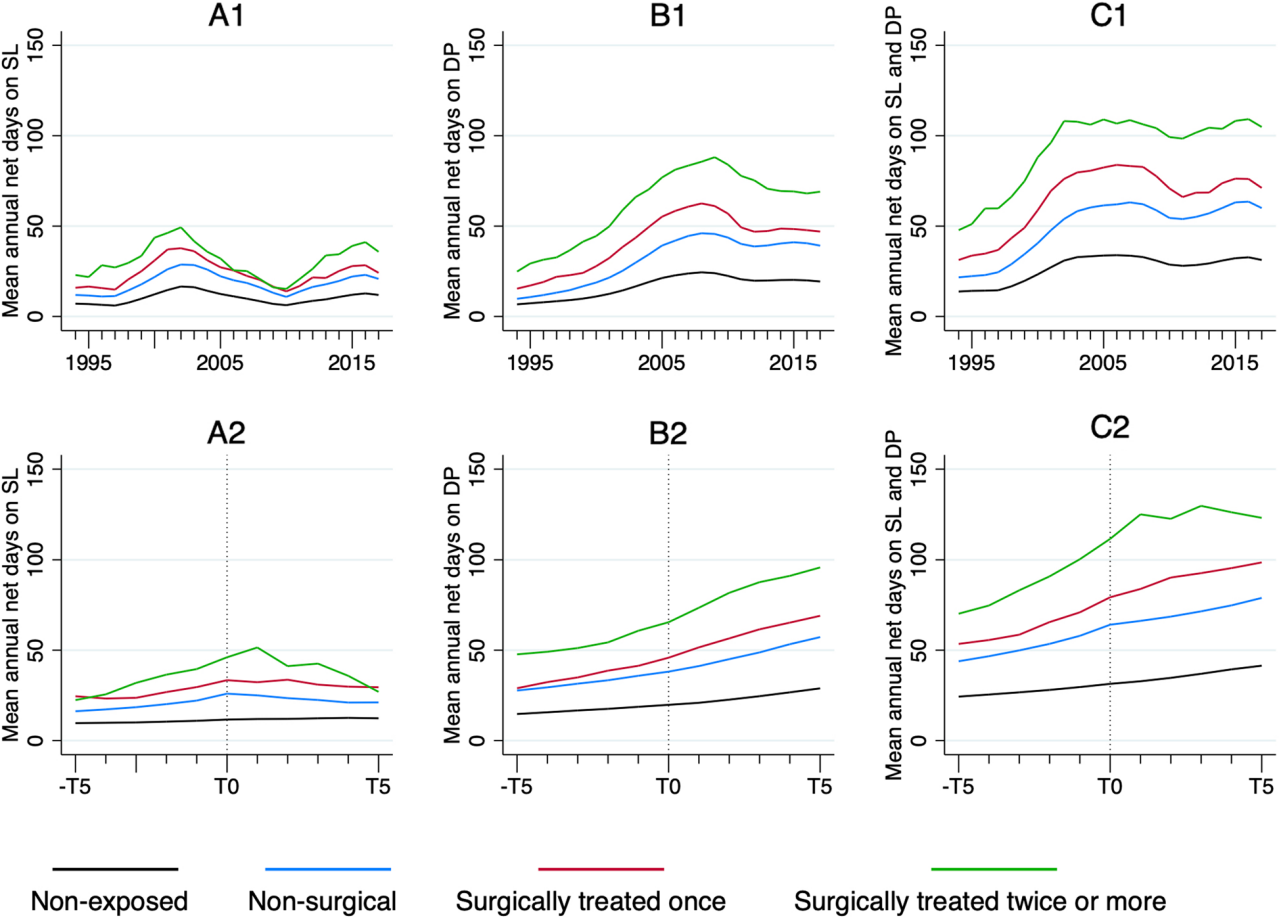
**A1** Annual days of sick leave in the cohorts 1994–2017, crude data. **A2** Annual days on sick leave five years before and after inclusion. Predicted averages, 95% CI drawn from generalized estimating equations and adjusted for all covariates. T0 is the time of inclusion. **B1** Annual days of disability pension in the cohorts 1994–2017, crude data. **B2** Annual days on disability pension five years before and after inclusion. Predicted averages, 95% CI drawn from generalized estimating equations and adjusted for all covariates. T0 is the time of inclusion. **C1** Annual days on sick leave and disability pension combined during the years 1994–2017, crude data. **C2** Annual days on sick leave and disability pension combined, five years before and after inclusion. Predicted averages, 95% CI drawn from generalized estimating equations and adjusted for all covariates. T0 is the time of inclusion

Mean annual net days on SL in all four groups between 1994 and 2017 are presented in Fig. 2 (A1). The mean annual net days of SL five years before (-T5) and five years after (T5) index date (T0) are presented in Fig. 2 (A2). In Table 3 and 4, a significant association between the exposed group and an increase in the outcome variable is shown in all exposed cohorts (*P* < 0.0001). The annual net days of SL are adjusted for age, education, sex, DEGURBA, and calendar year.

**Table 3:** Association between the cohorts and the outcome variables sick leave and disability pension and the association between the covariates time (year), sex, educational level, country of birth, degree of urbanization, age category and the outcomes. Inclusion year was also used as an adjustment variable. The model also includes the interaction between the cohorts and time, which is presented in Table 4

	**Sick leave**			**Disability pension**		
	β		95% CI	β		95% CI
**Cohort**						
General population (ref)	0	(ref)		0	(ref)	
Non-surgical	0.811	[0.808 0.814]		0.627	[0.625 0.630]	
Surgically treated once	0.980	[0.972 0.989]		0.814	[0.807 0.821]	
Surgically treated ≥ 2 times	1.259	[1.245 1.273]		1.113	[1.100 1.127]	
**Year**						
-T5	-0.184	[-0.186 -0.182]		-0.289	[-0.290 -0.289]	
-T4	-0.165	[-0.167 -0.163]		-0.227	[-0.228 -0.227]	
-T3	-0.144	[-0.146 -0.142]		-0.166	[-0.167 -0.166]	
-T2	-0.102	[-0.104 -0.101]		-0.116	[-0.116 -0.115]	
-T1	-0.059	[-0.060 -0.058]		-0.055	[-0.056 -0.055]	
T0 (ref)	0	(ref)		0	(ref)	
T1	0.026	[0.025 0.027]		0.053	[0.053 0.053]	
T2	0.015	[0.014 0.017]		0.101	[0.100 0.101]	
T3	0.021	[0.019 0.023]		0.145	[0.145 0.146]	
T4	0.010	[0.008 0.012]		0.182	[0.182 0.183]	
T5	-0.041	[-0.043 -0.039]		0.220	[0.220 0.221]	
**Sex**						
Male (ref)	0	(ref)		0	(ref)	
Female	0.630	[0.628 0.632]		0.420	[0.418 0.422]	
**Educational level**						
Primary and secondary school (< 9yrs) (ref)	0	(ref)		0	(ref)	
Upper secondary school (10–12 yrs)	-0.097	[-0.099 -0.095]		-0.671	[-0.675 -0.667]	
Post-secondary school (> 12 yrs)	-0.551	[-0.554 -0.548]		-1.671	[-1.684 -1.657]	
**Country of birth**						
Sweden (ref)	0	(ref)		0	(ref)	
Other Nordic countries	0.019	[0.015 0.023]		0.206	[0.201 0.210]	
Other European countries	0.051	[0.048 0.053]		0.345	[0.342 0.349]	
Outside Europe	-0.180	[-0.182 -0.177]		-0.053	[-0.056 -0.049	
**Degree of urbanization**						
Cities (ref)	0	(ref)		0	(ref)	
Towns and suburbs	0.085	[0.082 0.087]		0.045	[0.041 0.048]	
Rural areas	0.058	[0.056 0.060		0.047	[0.043 0.051]	
**Age category**						
23–25 (ref)	0	(ref)		0	(ref)	
26–30	0.542	[0.537 0.547]		-0.172	[-0.179 -0.165]	
31–35	0.867	[0.863 0.872]		0.043	[0.036 0.049]	
36–40	1.038	[1.033 1.042]		0.401	[0.395 0.408]	
41–45	1.086	[1.082 1.091]		0.760	[0.754 0.765]	
46–50	1.123	[1.119 1.128]		1.109	[1.103 1.114]	
51–55	1.196	[1.191 1.200]		1.444	[1.438 1.449]	
≥ 56	1.201	[1.196 1.205]		1.704	[1.699 1.710]	

*P* < 0.0001 for all values

**Table 4:** Continuation of Table 3, showing the interaction between time and exposure with days on sick leave and disability pension as outcome measures. The model also includes time (year), sex, educational level, country of birth, degree of urbanization, age category, inclusion year and cohorts

	Sick leave		Disability pension	
	β	95% CI	β	95% CI
Non-surgical*Year (ref general population)				
-T5	-0.289	[-0.294 -0.284]	-0.031	[-0.033 -0.029]
-T4	-0.247	[-0.252 -0.242]	-0.03	[-0.032 -0.029]
-T3	-0.199	[-0.204 -0.195]	-0.024	[-0.026 -0.023]
-T2	-0.152	[-0.156 -0.148]	-0.017	[-0.018 -0.015]
-T1	-0.101	[-0.104 -0.098]	-0.005	[-0.006 -0.005]
T0 (ref)	0	(ref)	0	(ref)
T1	-0.062	[-0.065 -0.059]	0.022	[0.021 0.023]
T2	-0.134	[-0.137 -0.130]	0.030	[0.029 0.031]
T3	-0.203	[-0.207 -0.199]	0.030	[0.029 0.032]
T4	-0.293	[-0.298 -0.288]	0.035	[0.033 0.037]
T5	-0.272	[-0.277 -0.266]	0.030	[0.028 0.032]
Surgically treated once*Year (ref general population)				
-T5	-0.127	[-0.140 -0.114]	-0.163	[-0.169 -0.157]
-T4	-0.199	[-0.211 -0.186]	-0.116	[-0.121 -0.111]
-T3	-0.203	[-0.214 -0.191]	-0.103	[-0.108 -0.099]
-T2	-0.118	[-0.129 -0.108]	-0.054	[-0.057 -0.050]
-T1	-0.065	[-0.073 -0.057]	-0.049	[-0.051 -0.046]
T0 (ref)	0	(ref)	0	(ref)
T1	-0.060	[-0.068 -0.052]	0.065	[0.062 0.067]
T2	-0.017	[-0.027 -0.007]	0.088	[0.085 0.091]
T3	-0.119	[-0.130 -0.107]	0.113	[0.109 0.116]
T4	-0.162	[-0.175 -0.150]	0.114	[0.109 0.118]
T5	-0.147	[-0.160 -0.134]	0.099	[0.094 0.103]
Surgically treated ≥ 2 times*Year (ref general population)				
-T5	-0.546	[-0.570 -0.522]	-0.028	[-0.037 -0.020]
-T4	-0.433	[-0.455 -0.411]	-0.061	[-0.068 -0.053]
-T3	-0.223	[-0.243 -0.204]	-0.081	[-0.088 -0.074]
-T2	-0.117	[-0.134 -0.101]	-0.072	[-0.077 -0.066]
-T1	-0.088	[-0.101 -0.075]	-0.020	[-0.024 -0.017]
T0 (ref)	0	(ref)	0	(ref)
T1	0.082	[0.070 0.095]	0.063	[0.059 0.067]
T2	-0.142	[-0.159 -0.126]	0.114	[0.109 0.119]
T3	-0.127	[-0.145 -0.108]	0.125	[0.119 0.131]
T4	-0.297	[-0.317 -0.276]	0.121	[0.114 0.128]
T5	-0.557	[-0.581 -0.533]	0.125	[0.117 0.133]

*P* < 0.0001 for all values

#### Disability pension

In Fig. 2 (B1), the mean annual net days on DP are presented for the years 1994–2017. There was as significant difference in the reliance on DP between the non-exposed group and the three exposed subgroups (Tables 3 and 4) (*P* < 0.0001). Figure 2 (B2) presents mean annual net days on DP from -T5 to T5 as described earlier. The model is adjusted for age, sex, education, DEGURBA, and calendar year.

#### Total days on sick leave or disability pension combined

During the 10-year follow period (-T5 to T5), the average of mean annual days of combined SL and DP was 32 in the comparison group, 61 in the non-surgical subgroup, 76 in the surgically treated once subgroup, and 104 days in the surgically treated twice or more subgroup. Figure 2 (C1 and C2) show the mean annual days of SL and DP combined during the years of follow-up as well in relation to the time of index (T0). All covariates are adjusted for in Fig. 2 (C2).

### Main results

Table 3 shows the results of the regression model with generalized estimating equations on net days of SL and DP including time (year) and the interaction between the cohorts and time, adjusted for age, sex, level of education, DEUGRBA, and calendar year. The table also depicts the association of the covariates with SL and DP. There was a positive association between the exposed subgroups and the outcome variables, as well as with being female, having lower education, being born outside of Sweden (except for being born outside of Europe) and increasing age.

Table 4 is a continuation of the model described in Table 3 and shows the results of said regression model and the interaction between the three exposed cohorts and time, in reference to the general population.

## Discussion

This study investigated whether there is a difference in the reliance on SL and DP between TMD-patients and the general population. The results indicate that patients diagnosed with TMD in a hospital setting and/or surgically treated for their condition are much more reliant on SL and DP benefits than the general population, even before they were introduced in the study. The increased reliance on these benefits is statistically significant as early as five years before the TMD-patients receive their diagnosis or first surgical treatment. The proportion of individuals that had ≥ 270 mean annual days of DP at T5 was 2–3 times more in the exposed cohort in comparison to the non-exposed cohort (7% versus 14/15/23%) (Table 2.) A similar ratio was shown regarding SL, where the exposed cohorts displayed an up to 8 times increased proportion of individuals receiving the benefits one year after first diagnose or first surgical intervention (T1) (1% versus 3/5/8%). It is however notable that the proportions on high number of SL decreases with time, and it is plausible to assume that TMD-patients for a shorter period in connection to receiving their diagnose or undergoing their first surgical event, are more reliant on SL than the average population, but over time become more reliant on more permanent means of reimbursements such as DP. DP is often a one-way event, but not always. According to current regulations individuals 19–29 years of age can be granted DP benefits when injury or disease cause reduced work capacity to full or partial extent and benefits can be granted for 1–3 years, or permanently (if there is no foreseen possibility to gain work capacity ever). Hence, the grade of reduced work capacity and DP are also re-evaluated. For individuals 30 years or older the benefit is permanent, with reduced work capacity 25–100%. Although the exposed cohort display a higher proportion of mean annual days of DP before T0, there is significant increase thereafter.

As TMD is a common disorder the impacts of such increased need of social security benefits are expected to be associated with high societal costs. A study on patients with persistent orofacial pain that had lasted for over 3 months showed not only that Graded Chronic Pain Scale (GCPS) was predictive of both patient and employer cost, but also that there was a difference of £2312 in employer costs between patient grading low vs high on the GCPS [22]. The gravity of pain according to GCPS has also been shown to affect both health care costs and Quality Adjusted Life-Years (QUALYs) [23]. To our knowledge there are no other studies that investigate the impact of TMD on SL and DP in Sweden, but there are studies on neighbouring symptoms such as cluster headache. Patients with cluster headache was shown to have a two-fold increase in SL (9.16% versus 17.30%) and increased DP with higher numbers in the female cohorts [24], similar to our findings. Although surgical treatment of TMD and its impact on SL and DP have also not been investigated earlier either, other surgical treatments methods have been evaluated using the same outcome measures. In a study on surgical treatment of rectal cancer from 2015, Chen et al. found that ten years after treatment the cumulative incidence of DP was 23.3% [25]. Another study on SL and DP and the association with Crohn’s Disease showed a prevalence of 19% for SL and 15% for DP. The study also showed that the patients with Crohn’s Disease had a total loss of workdays during the baseline year of 62 [26]. Our findings showed that women surgically treated for TMD on several occasions had an average of 70.6 days of DP over the entire 10-year follow up period. Muscular disorders and RA are strongly connected to TMD and a study on sick leave patterns in patients with musculoskeletal disorders from 2014 showed that during a two-year follow-up, patients with low back pain and myalgia used 26–27 mean days of SL, whereas disc disorders and rheumatoid arthritis used 147–150 mean days of SL [27]. A recently published seven-year follow-up study on SL of individuals suffering from chronic pain found that of the patients eligible for interdisciplinary treatment (IDT) 17% used SL five years before IDT, 48% used SL at T0, and 38% used SL at the end of follow-up [28]. The results of our study in comparison to other diseases are suggestive of the degree TMD-patients are suffering, however the comorbidities associated with TMD and their role in the need of SL and DP must be considered [29]. To what extent the results on the association between TMD and both SL and DP in our study can be explained by comorbidities and whether it is a true causal relationship cannot entirely be determined in this study and should be further investigated in future studies.

Generally, many surgical treatments are considered based on the patients’ probability of returning to work. A study evaluating SL after total knee or hip joint replacement due to osteoarthritis found very high percentages of SL in the surgically treated group shortly after treatment (89–90%) and lower levels at the two-year follow-up (9–17%) [30]. Similar outcome data are shown in studies of patients undergoing surgery for thumb carpometacarpal osteoarthritis [31]. The surgically treated groups in our study display high figures of SL and DP as late as five years after surgery, which indicates that TMD is a chronic and complex disorder that causes long-term work incapacity. If fewer days on SL or DP is the main outcome goal for surgical treatment, then surgical treatment is not a viable option. For TMD-patients, however, more factors are considered when evaluating the success of a surgical treatment, including function such as chewing, mouth opening ability, pain reduction, and quality of life. However, these measures of success were not possible to assess in this study. It is also interesting to see that TMD patients are reliant on both SL and DP as early as 5 years before they have their first time of diagnosis or surgical event (T0). Why this is can only be speculated, but as TMD patients in Sweden typically are treated with conservative treatment before they are referred for surgery, the high number of SL/DP in comparison to the general population might reflect the period of conservative treatment. TMD can debut in several different ways, and a well-known comorbidity of TMD is tinnitus [32]. Such symptoms might lead the patient to several other health care instances before meeting a physician or dentist that can diagnose TMD and subsequently treat the patient accordingly. Such a delay in diagnosing and treatment might be reflected in the high numbers of SL/DP in the non-surgical cohort, before inclusion in the study. There was a relatively high rate of patients treated surgically in this study (10%), compared to the 1% of the overall TMD-population that is expected to be referred for surgical treatment [6]. This is not unexpected and can be explained by the sampling of the exposed cohort. TMD patients diagnosed in a hospital setting as well as patients referred to OMFS are in fact a selected part of the total TMD population, of which individuals responding well to conservative treatment are not likely to be included.

There is a variance over time seen in Fig. 2 (A1, B1, C1) which influences the level of mean annual days on SL and DP in both the general population and the exposed cohorts, that can be explained by political decisions and changes in the benefit system. At the beginning of the twenty-first century, the regulations surrounding the eligibility of SL and DP became more lenient, although it was also stipulated that individuals with long-term SL would, to a larger extent than earlier, be considered for DP. This change in policy together with other system changes is hypothesized to have led to an increase in the termination of benefits but also an increased transition from SL to DP, predominately among women on long-term SL. In the second half of the first decade, the maximum amount of benefit per day was lowered, and the criteria for receiving SL was narrowed and regulated with certification requirements for certain diagnoses. The rules surrounding long-term sick leave of > 180 days also became stricter. Although changes in the system led to an increase in DP between 1998 and 2004, SL started to decline in 2002 and DP followed the pattern in 2004 with an all-time low for both SL and DP in 2010. The decrease has been partially explained by more strict and systematic assessment of eligibility, but the mechanisms are not completely understood [33, 34]. The transition of long-term SL into DP is also seen in our study population, described in Table 2. The exposed cohorts display a higher percentage of individuals on SL at T0, that is gradually decreasing to T5, whereas DP is instead increasing from T0 to T5.

### Strengths and limitations

This study has several strengths. No other study has been able to present data on sickness insurance benefits among TMD-patients in a population-based study. The cohorts are uniquely large thanks to the intricate and exceptional registries in Sweden. The registry data have no loss to follow-up and is registered prospectively with no risk for recall bias. Because the data are largely typical for the Swedish and perhaps the Nordic countries with similar social benefit systems, the conclusions drawn from this study regarding the possibilities of SL and DP benefits among TMD-patients are most probably restricted to the Nordic countries. Regardless of the differences in the social insurance systems; the results of this study illustrate very well how this patient group is extremely vulnerable and unable to lead a life with full working capacity.

The largest limitation in this study is that a registry is a blunt instrument for measuring a heterogenic patient group such as TMD with respect to diagnosis. For example, there is no measure to confirm that the ICD-10 code K07.6 is indeed given performing the use of DC/TMD or any other established instrument of diagnosis. The registry, however, is very useful for identifying subjects who have received surgical treatment for these conditions. Therefore, the conclusions from this study must be taken cautiously with respect to the non-surgical group. It is also hard to determine whether subjects in the non-surgical group will receive surgical treatment after the inclusion time, meaning that there is a risk of misclassification of exposure-level in the non-surgical cohort. TMD-patients are known to display several comorbidities; as this study does not consider other illnesses or disorders, we cannot reject the possibility that part of the difference in the outcome measures is due to unknown comorbidities that are part of the causal pathway for developing TMD and act as confounding factors. In addition, it is important to consider the fact that in Sweden the absolute largest proportion of TMD-patients are diagnosed and treated within the dental care system and will never be diagnosed in a hospital setting. Many of the patients seeks medical care if the symptoms are intensely pronounced and quite often miss the connection to dental care because the experienced issue is not clearly related to the teeth. Also, the patients may choose to visit the emergency hospital, in case of pronounced symptoms with sudden onset and with large impact on the daily life activities. A contributing factor may also be that, in Sweden health care is almost free of charge while dental is expensive for the patients. Considering that the non-surgical cohort within this study only compile of the fraction of the entire TMD-population, the conclusions from this study might be inferred on a subset of the TMD cases within this very large and heterogeneous patient group.

## Conclusion

Individuals diagnosed with TMD, both non-surgically as well as surgically treated, display a dependence on income from SL and DP benefits that is 2–3 times higher than the general population. This reliance does not seem to decrease with time as one might expect and occurs even before diagnosis. The results of this study indicate that TMD-patients as a group, whether they need surgical treatment or not, are more reliant on social benefits than a non-exposed comparison cohort and are perhaps a more vulnerable group than previously thought during and after treatment. It is however of importance to acknowledge that the non-surgical group constitute a small portion of the entire TMD-population and the conclusions drawn on the subgroup must be taken with some consideration.

## Data Availability

The data that support the findings of this study are available from the National Board of Health and Welfare and Statistics Sweden, but restrictions apply to the availability of these data, which were used under license for the current study and therefore are not publicly available. Data are, however, available from the authors upon reasonable request and with permission of said registry holders and Ethics Board.

## Abbreviations

TMD: Temporomandibular Disorders
TMJ: Temporomandibular Joint
DC/TMD: Diagnostic Criteria for Temporomandibular Disorders
OMFS: Oral and Maxillofacial Surgeon
NPR: National Patient Registry
TMJD: Temporomandibular Joint Disorders
SL: Sick leave
DP: Disability pension
SWEREG-TMD: Swedish National Registry Studies for Surgically Treated TMD
NBHW: National Board of Health and Welfare
TPR: Total Population Registry
LISA: The Longitudinal Integrated Database for Labour Market and Health Insurance Studies
SCB: Statistics Sweden
PIN: Personal Identification Number
ICD: International Classification of Diseases
DEGURBA: Eurostat’s Degree of Urbanisation
